# Practical Notes on Diagnosis and Treatment

**Published:** 1907-04-06

**Authors:** 


### Practical Notes on Diagnosis and Treatment.

**Glycosuria.**

Patients who have once had sugar in their urine should be carefully warned, after its cessation, never to touch sugar in any form; and in such matters as bread, potatoes, fruit, etc., to be exceedingly moderate—in fact, never to indulge in any two of these at one and the same meal.—*Dr. Chas. H. Ralfe.*

**Anal Fissure.**

Allingham has strongly advocated the following ointment; he has known it produce many cures without other treatment:—Calomel 4 grains, opium in powder 2 grains, belladonna extract 2 grains, elder flower ointment 1 dram. To be applied frequently. Another ointment for the same purpose is:—Lead acetate 10 grains, zinc oxide 10 grains, prepared calamine 20 grains, benzoated lard half an ounce.

**Treatment of Hæmorrhoids.**

In my experience nothing has afforded such comfort to patients suffering from piles as the daily use of a small enema. I generally prescribe not more than three-quarters of a pint of tepid water, to be gently injected into the bowel every morning. The result is that the act of defecation is accomplished extremely quickly, and the motion is passed with the least possible injury to the anus. I have not seen any ill-effect from the daily use of an enema of the size I have mentioned, continued even over a long period; on the contrary, I have known patients who have suffered considerably for years entirely relieved by this treatment alone. A soft flexible tube should be fitted over the ivory or metal nozzle of the enema apparatus.—*Mr. A. Pearce Gould.*

**Malignant Disease of the Spine.**

Malignant disease of the spinal column as a secondary consequence of malignant disease of the breast is more common than any other sort of cancer of the spine.—*Sir Wm. H. Bennett.*

**Sympathetic Ophthalmia.**

Whenever symphathetic ophthalmia becomes developed we should carry the sustained administration of mercury by inunction, together with the internal administration of quinine, to the fullest limits which the condition and idiosyncrasies of the patient will allow.—*Mr. Brudenell Carter.*

**Turpentine in Puerperal Septicæmia.**

Turpentine is of special importance in all cases of puerperal septicæmia in which its use may be tolerated by the stomach and bowels. When given in ʒss doses every third or fourth hour, and when not contra-indicated by gastro-intestinal complications, it has a marked influence in relieving meteorism, stimulating the system, and improving the discharges of the puerperal patient.—*Dr. More Madden.*

**Mercury in Tabes Dorsalis.**

I am no believer in the necessity for vigorous courses of mercury in the treatment of ordinary tabes. Should symptoms of the disease appear within a short period of the primary infection, I would certainly push that remedy, but not when there is an intervening long period of good health. In the latter cases I always give mercury in small doses, but it is in combination with wine and a liberal diet; and my chief insistence is upon rest in general, and especially in reference to sexual matters.—*Mr. Jonathan Hutchinson.*

